# Feature level quantitative ultrasound and CT information fusion to predict the outcome of head & neck cancer radiotherapy treatment: Enhanced principal component analysis

**DOI:** 10.1002/mp.18078

**Published:** 2025-09-03

**Authors:** Amir Moslemi, Aryan Safakish, Lakshmanan Sannachi, David Alberico, Gregory J. Czarnota

**Affiliations:** ^1^ Physical Sciences, Sunnybrook Research Institute Sunnybrook Health Sciences Centre Toronto Ontario Canada; ^2^ Department of Radiation Oncology Sunnybrook Health Sciences Centre Toronto Ontario Canada; ^3^ Department of Physics Toronto Metropolitan University Toronto Ontario Canada; ^4^ Department of Medical Biophysics University of Toronto Toronto Ontario Canada

**Keywords:** head and neck cancers, information fusion, PCA, quantitative CT, quantitative ultrasound spectroscopy, radiomics

## Abstract

**Background:**

Radiation therapy is a common treatment for head and neck (H&N) cancers. Radiomic features, which are determined from biomedical imaging, can be effective biomarkers used to assess tumor heterogeneity and have been used to predict response to treatment. However, most studies employ only a single biomedical imaging modality to determine radiomic features.

**Purpose:**

The objective of this study was to evaluate the effectiveness of radiomic feature fusion, combining quantitative ultrasound spectroscopy (QUS) and computed tomography (CT) imaging modalities, in predicting the outcomes of radiation therapy for H&N cancer prior to start.

**Method:**

An enhanced version of principal component analysis (EPCA) was proposed to fuse 70 radiomic features from CT and 476 radiomic features from QUS in order to predict the response to radiation therapy in patients with H&N cancers (partial response vs. complete response). EPCA is a PCA method with Hessian matrix regularization and $l_{2,1-2}$ ‐regularization, and was proposed here for information fusion at a feature level. Leave‐one‐patient‐out methodology with bootstrap was applied to conduct train‐test analysis and fused features were used to train two (support vector machine (SVM) and k‐nearest neighbor (KNN)) classifiers to build a predictive model in order to predict response to treatment for patients with H&N cancers. Five‐fold (5) cross validation was applied on the training set to tune the hyperparameters of SVM and KNN classifiers. Consequently, the performance of classifiers was evaluated by examining accuracy (ACC), F1‐score (F1), balanced accuracy (BACC), Sensitivity (S_n_), and Specificity (*S*_p_) metrics. Additionally, a two‐sided *t*‐test was applied to the top principal components derived from EPCA methodology in order to assess the statistical significance of the selected components. The proposed method developed here was compared with minimum redundancy maximum relevance (mRMR) feature selection, conventional PCA, kernel PCA, autoencoder, and canonical correlation analysis (CCA). Additionally, we compared proposed EPCA with robust PCA and $l_{2,1}$ ‐norm constrained graph Laplacian PCA.

**Results:**

Seventy‐one (*n* = 71) (66 male (93%) and female (7chmch%)) H&N cancer patients were recruited with bulky metastatic neck lymph node (LN) involvement. Patients had a mean age of 59 ± 10 and 25 (35.2%) were complete responders and 46 (64.8%) were partial‐responders. In terms of predicting responses, the EPCA‐SVM classifier had better performance than EPCA‐KNN, and achieved 79±2% sensitivity, 84±2% specificity, 82 ±2% accuracy, 81±  2% balanced accuracy, and 82 ±2% area under curve (AUC). Results demonstrated the effectiveness of the proposed method with superiority over mRMR feature selection, conventional PCA, kernel PCA, autoencoder, and CCA methods. Using an ablation study, EPCA was compared with robust PCA and $l_{2,1}$ ‐norm constrained graph Laplacian PCA. Results supported the superiority of EPCA over rPCA and $l_{2,1}$ ‐norm constrained graph Laplacian PCA. Three principal components were statistically significant. Additionally, we compared the proposed method with the use of QUS and CT as individual imaging modalities. The results demonstrated the effectiveness of feature‐level fusion in enhancing prediction accuracy.

**Conclusion:**

The results demonstrated that the proposed predictive model is able to predict a binary H&N cancer treatment outcome, feature level fusion of CT and QUS radiomics has superiority over single imaging modality and EPCA is an effective approach to fuse the features.

## 1 INTRODUCTION

Head and neck (H&N) cancer includes oral cavity, pharynx, larynx, paranasal sinuses, nasal cavity, and salivary gland cancers.^1^ Collectively these are ranked as the 6th most frequently encountered cancer type.^2^ Approximately 930 000 new cases are diagnosed every year based on the World Health Organization report^3^ with 90% of H&N cancers are categorized as squamous cell carcinoma.^4^ Tobacco,^5^ alcohol,^6^ p53 and p16 mutations,^7^, ^8^, ^9^ and human papillomavirus (HPV)^10^ are factors that increase the risk of developing H&N cancer. Although distant metastasis is rare for H&N cancer, regional lymph node involvement is frequently found at the time of diagnosis often becoming bulky and causing local problems.^3^ The 5‐year disease‐related mortality is affected by the stage and location of tumors.^11^

Surgery, systemic therapy, radiotherapy, and combinations thereof are used for treatment based on the stage of H&N cancer. Standard radiotherapy as a primary treatment modality for H&N cancer can include doses such as 70 Gy in 33‐35 fractions to gross disease, and 56 Gy in 33‐35 fractions delivered to regions at risk for microscopic spread, respectively.^12^ Treatment outcomes can be improved by new high‐precision techniques such as intensity‐modulated radiation therapy (IMRT) and volumetric‐modulated arc therapy (VMAT). Despite such technological advances, and even with the use of concurrent chemotherapy for select patients, complete response cannot be easily achieved for patients.^13^

There is evidence to support a correlation between tumor heterogeneity—as a feature of aggressive disease—and the effectiveness of the response to treatment.^14^ Studies have indicated that the effectiveness of the response to treatment can be reduced by tumor heterogeneity.^15^ Biological resistance to treatment emerges often with aggressive disease and is reflected in heterogeneity of tumor imaging modalities including magnetic resonance imaging (MRI), computed tomography (CT), positron emission tomography (PET), and quantitative ultrasound spectroscopy (QUS) can be employed for head and neck cancer, planning treatment, and assessing post‐treatment response. Versions of each of these imaging modalities have been investigated in monitoring H&N treatment response. Imaging using ultrasound is easy, inexpensive, and portable. It allows for rapid scan acquisition with consistent and reliable patient participation in care. B‐mode ultrasound imaging is a well‐established approach for H&N cancers, particularly in identifying lymph node metastasis.^16^ It can detect sub‐clinical neck nodes, providing valuable information about the progression of cancerous cells.^17^ QUS processes raw radiofrequency (RF) data, offering an advantage over B‐mode imaging by extracting more detailed information about tissue characteristics.^18^ Additionally, the elastic properties of tissue at the microcellular level provide insights into biological characteristics that can be used to interpret tumor response. Spectral parameters extracted from QUS reflect cellular density, scatterer size, and tissue organization, all of which are associated with biological outcomes.^19^ Ongoing changes in the elastic properties of tissue, associated with cell death, can be tracked using QUS during treatment. This enables earlier detection of treatment response compared to conventional imaging techniques.^20^ Ultrasound imaging not only is relatively inexpensive rather than PET and MRI, but also it does not require contrast agents.^20^

Nevertheless, the resolution of these imaging techniques is not sufficient to provide information at the level of individual tumor cells. To this end, radiomics features are often determined in order to quantify tumor features non‐invasively in aggregate and at various structural scales^21^ for various purposes. The effectiveness of QUS in predicting treatment response for head and neck malignancies, both before and during treatment, has been demonstrated. For example, treatment outcomes have been predicted using pre‐treatment texture‐derivative‐based QUS radiomics,^22^ and treatment response has been assessed using QUS delta‐radiomics in patients with H&N cancers.^23^ In contrast to conventional gray‐scale sonography, QUS is independent from user and machine settings.^20^

Machine learning (ML) techniques have been widely applied in cancer research to predict responses to treatment. Safakish et al.^24^ extracted radiomic features and texture features from QUS images for patients with H&N cancer and applied different ML classifiers to predict partial and complete responders for radiation therapy treatment. Zhang et al.^25^ used CT‐based radiomic features and multivariable logistic regression to predict responses to chemotherapy in locally advanced head and neck squamous cell carcinoma. Ren et al.^26^ extracted radiomic features from T2‐weighted and T1‐weighted MRI images and utilized least absolute shrinkage and selection operator (LASSO) logistic regression to classify stages of head and neck squamous cell carcinoma. Whereas Mes et al.^27^ employed MRI radiomic features to predict outcomes of head and neck squamous cell carcinoma, Starke et al.^28^ determined radiomic features from both CT and FDG‐PET images to predict outcomes of locally advanced head and neck squamous cell carcinoma using Cox proportional hazards.

All of above studies considered radiomic features from only one imaging modality. Utilizing information from several imaging modalities can potentially lead to improvements in outcome prediction for H&N cancer.^28^ Although research^29^ has considered two imaging modalities, the determined features from CT and PET were naively concatenated, which can lead to a loss of correlation between imaging modalities. Nevertheless, the performance of ML classifiers can be improved by fusing information from different imaging modalities

Information fusion can be categorized into pixel level, a feature level, and at a decision level. In the medical domain, information fusion can be challenging due to misregistration, which reduces the performance of ML classifiers.^30^ However, image fusion at the feature level does not suffer from misregistration, and the performance of ML classifiers is not affected.^31^ Principal component analysis (PCA) is a dimensionality reduction technique that seeks directions to maximize the variance of data. PCA has been widely applied for information fusion^32^, ^33^, ^34^ but has two drawbacks that affect the performance of this technique. Specifically, PCA is not able to preserve geometric and topological information of data, and it can be considerably sensitive to noise and outliers.^35^ Although robust PCA was introduced to circumvent the outlier problem, it utilized an $l_{1}$ ‐norm to sparsity solution which is not particularly sparse in comparison with $l_{p}$(0<p<1) and used a nuclear norm to extract low‐rank properties which is not as good as a Shutten‐p norm (0<p<1).^36^ Although a $l_{p}$ ‐norm constraint with 0<p≤1 was employed to sparsify the solution of PCA, the geometry of data in the low‐dimensional manifold is not considered and subsequently the topology of data in subspace is not preserved.^37^ To this end, a Laplacian graph matrix was added to preserve geometrical information of data in representation space with RPCA.^37^

The primary challenges in the aforementioned studies include the following:

1. using a single imaging modality
2. the $l_{1}$ ‐norm lacks sufficient sparsity and serves as only a relaxed version of the $l_{0}$ ‐norm
3. the $l_{p}$ ‐norm (0<p≤1) is neither convex nor Lipschitz continuous.
4. the Laplacian graph matrix suffers a rich null space (Cannot preserve the geometry of data efficiently).

**FIGURE 1:**
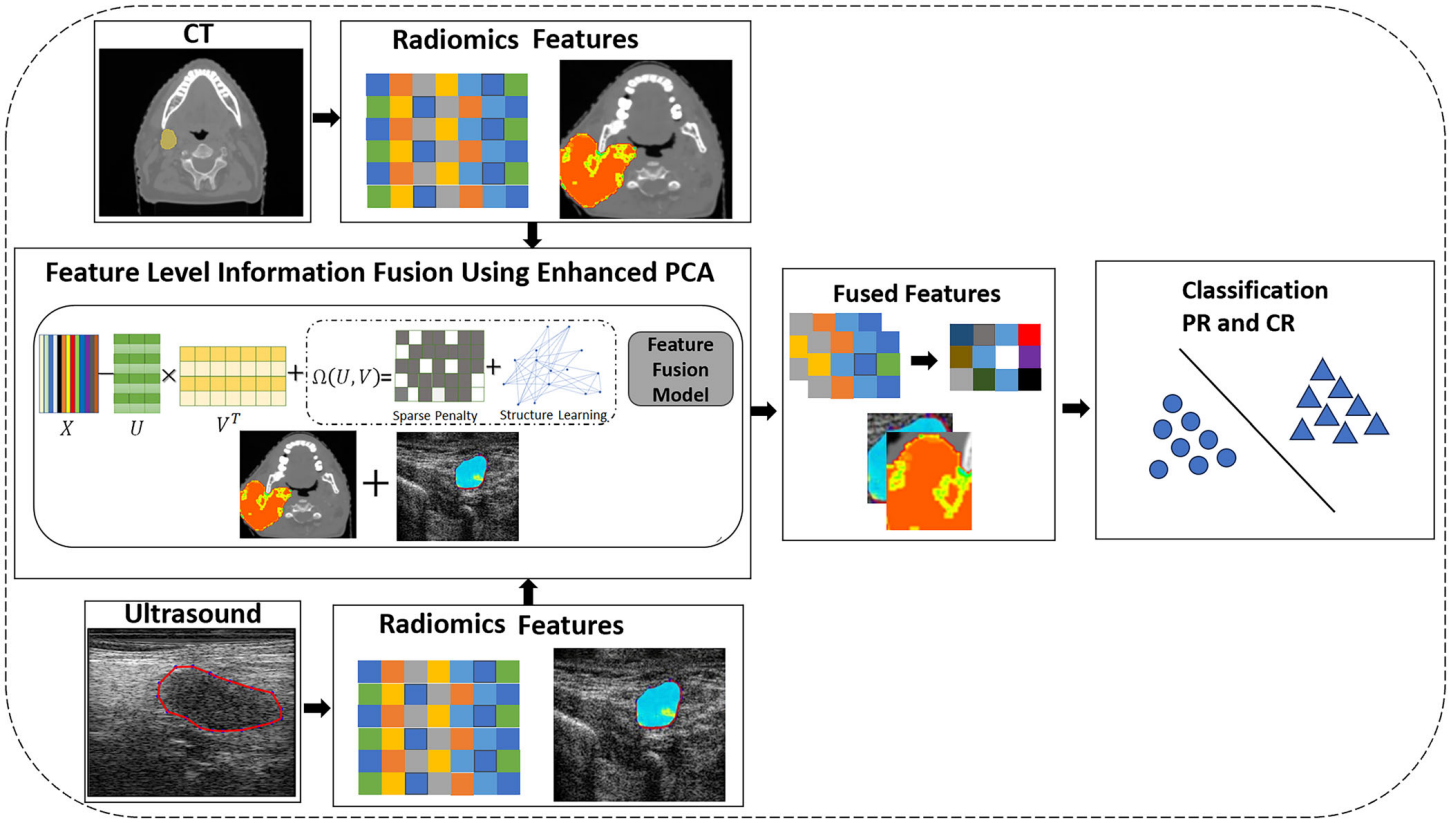
Proposed methodology. The radiomics features are extracted from the CT and QUS images and then EPCA is applied to fuse these features. Finally, a classifier is trained by fused features to discriminate complete responder and partial responder patients.

In order to address all above‐mentioned challenges, an enhanced PCA method is proposed here to fuse the radiomics features of CT and QUS in order to predict response to treatment of H&N cancer patients. The study here added Hessian graph regularization, which has a more extensive null space than a Laplacian graph matrix, in order to preserve the geometry of data.^38^ Additionally, an $l_{2,1-2}$ norm constraint was used to suppress noise and generate sparse solutions, which is Lipschitz continuous.^39^ Figure 1 represents the proposed method.

In the management of head and neck cancer CT and MRI are not used clinically for the prediction of response to chemoradiotherapy and radiotherapy. CT and MRI are not used for this clinically. Rather, CT and MRI scans are typically obtained 3 months after the completion of a course of chemoradiotherapy and radiotherapy. They are also obtained up front for diagnostic purposes but not used to predict or monitor responses during treatment. Here we investigate the fusion of functional imaging (QUS)) with diagnostic grade CT—which we process to also attempt to extract useful prognostic features.

Specifically, the objective of study here was to build a predictive model based on machine learning to predict responses to radiotherapy treatment for H&N cancer using information fusion of CT and QUS imaging modalities at a feature level. The working hypothesis of this work was that fusing radiomics features of CT and QUS can improve classifier performance in predicting response to treatment for patients with H&N cancer a priori to the start of treatment.

## 2 MATERIALS

### 2.1 Study procedure and treatment

This study was approved by the institutional research ethics board (REB 3047 – Sunnybrook Research Institute). Enrolled patients (n=71) were scheduled to undergo radiotherapy (XRT) encompassing biopsy‐confirmed H&N cancers with pathologically involved index lymph nodes (LNs). Subjects were recruited with informed consent to participate in acquisition of ultrasound scans at various times throughout the XRT regimen, in addition to the other necessary imaging acquisition required for treatment‐planning and dosimetric calculations (CT scan). Index LNs (typically, nodal sizes are reported as a 2D in‐plane measurement (maximum and minimum) were considered pathological based on a criterion of whether the smaller of the two reported measurements, the “short axis”, measured ≥15 mm, as observed with a CT scan. As per standard of care protocols, patients were treated with curative intent radiotherapy with or without concurrent chemotherapy. Patients received 70 Gy to high‐dose, 63 Gy to intermediate‐dose, and 56 Gy to low‐dose targets in 33 fractions using IMRT or VMAT techniques according to institutional standard of care. Patients receiving systemic chemotherapeutics were treated with either cisplatin, carboplatin, or a combination of both, and received treatment concurrent with their radiotherapy.

### 2.2 Data acquisition

#### 2.2.1 Ultrasound

Recruited patients agreed to undergo ultrasound scans lasting between 20 and 45 min each time. Guided by oncologist and radiologist‐input, the largest LN was scanned by trained technicians to capture the entire LN volume along 256 lateral scan lines (in‐plane; 3.8 cm lateral field of view and maximum axial depth of 5 cm). The acoustic focus was adjusted manually based on the depth of each individual's LN with an average depth of 1.75 cm. B‐Mode image data and associated digitized radio frequency (RF) signals were acquired the latter with a digital sampling rate of 40 MHz using an Ultrasonix Med. Corp.) device with linear transducer (L14‐5/38 Linear 4D, Ultrasonix) with a central frequency of approximately 7.5 MHz. In this data, the interest was in gaining a priori insights regarding retrospective treatment outcomes and thus for this particular study, the baseline scans obtained before treatment were utilized. The largest lymph node was identified with oncologist. A ‘baseline’ ultrasound scan of the largest involved lymph node was done for each patient, 2 weeks prior to starting (chemo)radiation, at the time of treatment‐planning for XRT using CT simulation. Only prior to treatment ultrasound images were used to build a predictive model for the response of treatment prediction.

#### 2.2.2 CT

Since CT scanning is a necessity for treatment planning, subjects did not have to undergo any additional burden for participation in this portion of the study. Treatment planning CT scans, segmentations, and transformation matrices were retrieved from institutional database as DICOM files. DICOM files were opened with the open‐source software 3D‐slicer and it was ensured that the treatment planning segmentations were accurately registered to the CT image anatomy with the transformation matrix. Target LN region of interests (ROIs) were isolated and saved as .nrrd files while the CT scans were saved as .nii file. The CT simulator machine a 32 slice Philips wide bore device (Philips Brilliance CT Big Bore scanner) with a slice thickness of 1.5 mm. CT Bore size (gantry aperture diameter) was 85 cm with two collimations 16 × 1.5 cm and 16 × 0.75 cm for use. The true field of view (FOV) was 60 cm with a 120 s maximum scan time. Focal spot was 1 mm, convolution kernel was standard and pitch was 0.442. Energies available for use were 120 or 140 kVp and patients received a 80 mL dose of OMNI350 (manufacturer) iodinated contrast using an injection rate of 2 mL/s. Patients were imaged 45s after injection.

#### 2.2.3 Radiomics features

After the acquisition of US data, in‐house MATLAB software was used to view the B‐mode ultrasound slices and highlight the boundaries of the LN to create a spatial ROI matrix for 6 equally spaced slices spanning the entirety of the ROI. Next, utilizing stored RF data, QUS spectra were evaluated by following methods established by Lizzi et al.^40^ which includes; (i) applying a Hamming window to suppress spectral side lobes, then (ii) a fast Fourier transform (FFT) to determine the frequency component of the signal which is followed by (iii) squaring the magnitudes of the resultant spectra to arrive at an average power spectrum, and finally (iv) arriving at the normalized power spectrum by dividing with the average power spectrum of a tissue‐mimicking phantom with known acoustic properties with the goal of minimizing various frequency dependent transfer functions and beam forming effects associated with the transducer.^40^

**FIGURE 2:**
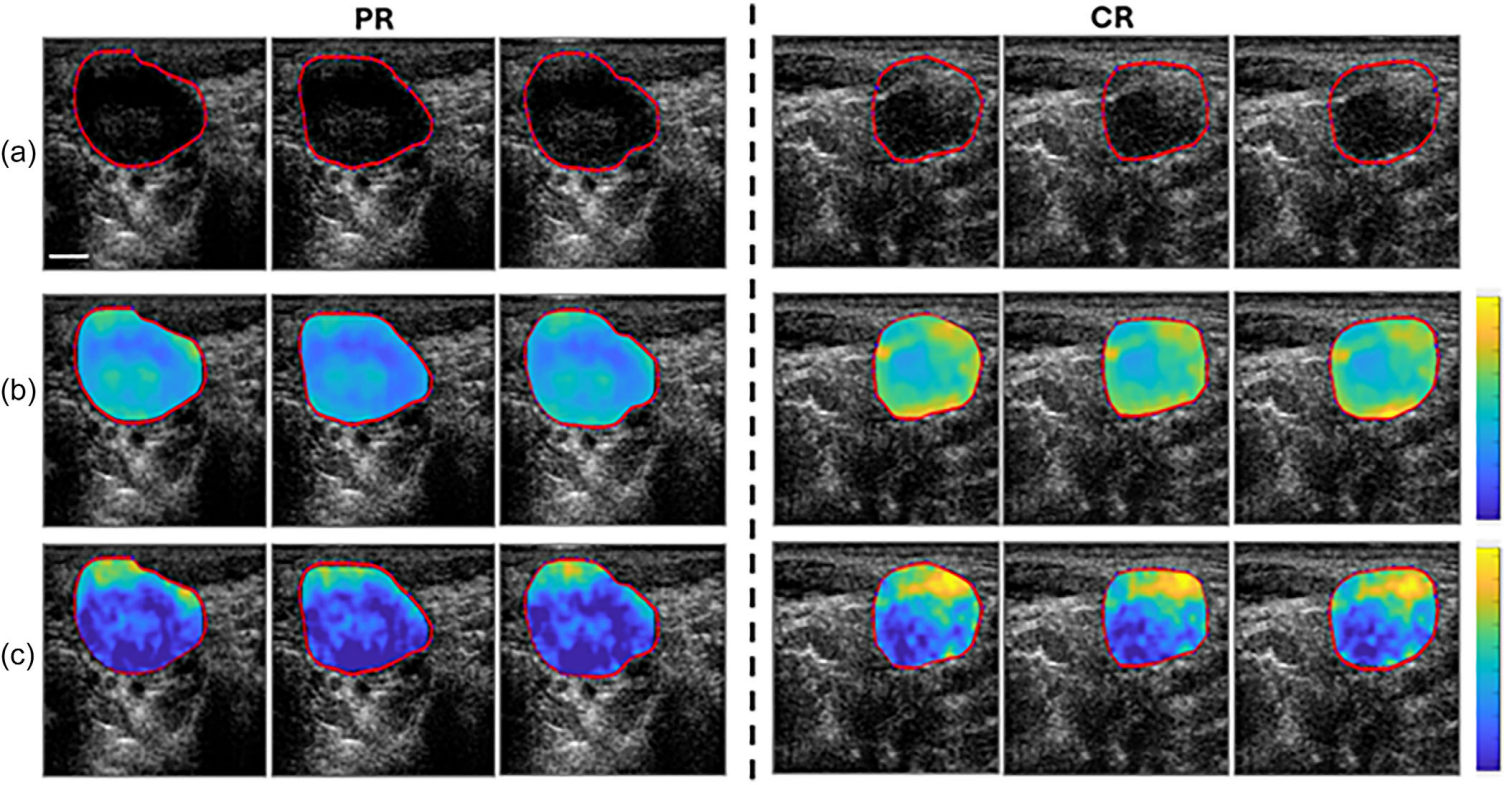
Representative QUS parametric maps of three CR patients (On the left) and three PR patients (On the right). (a) Presents the B‐mode ultrasounds with ROIs. (b) Mid‐Band fit parametric maps (range from −10 to 40 dB). (c) Spectral intercept parametric maps (range from 10 to 50 dB). Scale bar is 5 mm.

Next, within a 6 decibel(dB) window centered on the transducer's central frequency, linear regression analysis was performed on the quasi‐linear normalized power spectrum in accordance with previously established theoretical frameworks.^41^ From the best fit line, mid‐band‐fit (MBF), spectral slope (SS), and spectral intercept (SI) were computed,^41^ along with two back scatter coefficient parameters average scatter diameter (ASD) and average acoustic concentration (AAC), using both fluid filled (Anderson model),^42^ and a Gaussian model.^43^ The seven aforementioned QUS parameters were calculated using a sliding window technique with a 2×2  mm window size with 92% overlap in order to create QUS parametric maps. Figure 2 shows representative QUS parametric maps for three complete responder (CR) patients and three partial responder (PR) patients.

For each subject, using Pyradiomics,^44^ an open‐source Python (Python Software Foundation) package was used to “mine” 2D gray‐level co‐occurrence matrix (GLCM), gray level dependence matrix (GLDM), gray level run length matrix (GLRLM), and gray level size zone matrix (GLSZM) radiomics features from each normalized QUS parametric map LN ROI to arrive at a total of 476 QUS radiomics features. This process was carried out for each of the six slices and averaged to quantify the textural feature.

**FIGURE 3:**
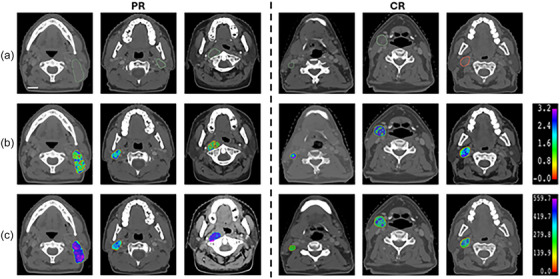
Representative CT radiomics features of three CR patients (On the left) and three PR patients (On the right). (a) Presents the CT image with ROIs. (b) GLDM‐ dependence entropy ([0–3.5] range). (c) GLCM cluster shade ([0–600] range). Scale bar is 5 mm.

Next, 2D CT radiomics features were determined from LN ROIs of axial slices of treatment planning CT scans and averaged. Note that in this study QUS features were “mined” from various QUS parametric maps, which have been shown to characterize tissue microstructures,^45^, ^46^ whereas the CT radiomics features were mined directly from the treatment‐planning CT images themselves and thus reflect on relationships of neighboring pixels’ normalized Hounsfield Units intensities. Subsequently, 70 CT radiomics features were determined. Figure 3 shows the CT radiomics features of three CR patients and three PR patients.

## 3 METHODS

### 3.1 Background

$X\in R^{p\times n}$ was input data (combination of QUS and CT radiomics features) such that p and n are the number of features and samples, respectively.

#### 3.1.1 PCA

The aim of PCA is to find optimal low‐dimensional subspace with all essential information or original data X.^47^ PCA looks those directions which maximize the variance of data. Principal directions and principal components are two variables of PCA which can be derived from following optimization problem:

(1) $$\min_{U,V}\frac{1}{2}{∥X-UV^{T}∥}_{F}^{2}\;\;\;s.t\;V^{T}V=I$$

where $U\in R^{p\times k}$ is the principal directions, $V^{T}\in R^{k\times n}$ is the optimal low‐dimensional (k ‐dimension) new subspace, and I is identity matrix.

### 3.2 Manifold embedding using graph learning

**FIGURE 4:**
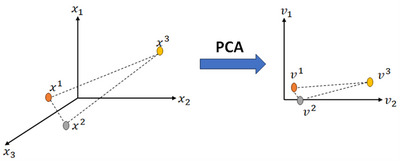
Manifold Embedding: $x^{1}$, $x^{2},$ and $x^{3}$ are three samples in original space X and $v^{1}$, $v^{2},$ and $v^{3}$ are corresponding samples in subspace V. $v^{1}$, $v^{2},$ and $v^{3}$ are corresponding of $x^{1}$, $x^{2},$ and $x^{3}$ in subspace.

PCA can identify an approximate set of fundamental vectors when dealing with data that primarily resides within a linear manifold.^47^ Taking into account the local invariance of the inherent geometric structure within the data distribution, recent research in the field of non‐linear manifold learning theory has highlighted the popularity of graph Laplacian embedding.^48^ The fundamental assumption of local invariance assumes that if two points (samples) are proximate within the original data distribution's intrinsic geometry, their representations in the new coordinate system will also be proximate to each other. The local geometric structure can be effectively represented through a nearest neighbor graph constructed on a scatter of data points. Loosely speaking, if $x_{1}$ and $x_{2}$ are two close samples in X, corresponding of $x_{1}$ and $x_{2}$ in V (subspace) must be close to each other. Figure 4 shows an example for three samples in high dimensions and subspace.

Because of the Laplacian's focus on addressing first derivative issues, its solution tends to exhibit a bias toward constancy, resulting in limited extrapolation capabilities and an insufficiency in capturing comprehensive structural information. To overcome these challenges, we suggest the incorporation of Hessian regularization within the framework of principal component analysis.^49^, ^50^, ^51^ The Hessian matrix possesses a more extensive null space, and by leveraging the geodesic function within this null space, it can effectively harness the intrinsic local geometry of the data manifold. This approach accurately reflects the positional relationships between samples and yields a wealth of structural information, enriching the low‐dimensional representation with a more abundant set of graph‐related insights.^38^

### 3.3 Hessian regularization

The Hessian regularization function is utilized on the data manifold to retain the geometric details of individual instances. In this research, we adopt the notion of the generalized uncorrelated constraint from Hessian regularization, originally devised for semi‐supervised feature selection.^49^, ^50^

The mathematical proof Hessian graph for structure learning can be found in supplementary file S‐Hessian. Hessian matrix construction has four steps which are summarized as follows:

1. Construct a neighbor matrix using KNN (K‐nearest neighbors).
2. Obtain a tangential coordinate system by applying singular value decomposition (SVD) on neighbor matrix.
3. Estimation of the Hessian energy using least square technique.
**4**. Calculate structure learning regularization using $\Omega \left(V\right)=V^{T}HV$. Where H is Hessian matrix and $V^{T}$ is the optimal low‐dimensional. The Hessian energy enables functions that extrapolate beyond the range of the training outputs. It favors functions that exhibit linear variation along the manifold, known as geodesic functions, which will be defined later.^47^ The proposed Hessian energy is inspired by the Eells energy for mappings between manifolds,^50^ which includes as a special case the regularization of real‐valued functions on a manifold. Conceptually, it bears similarities to the operator introduced in Hessian eigenmaps.^51^, ^52^ Details of Hessian matrix calculations and structure learning regularization can be found in supplementary file S‐Hessian.

### 3.4 Sparse regularization: $l_{2,1-2}$ ‐norm

In order to enhance the sparsity of feature expression in feature fusion, $l_{2,1-2}$ ‐norm regularization was used. In sparse analysis, $l_{1}$ was introduced as an alternative to $l_{0}$, but nonconvex (concave) quasi $l_{p}$ (0<p<1) norm, with the intention of promoting sparsity in the solution, was introduced.^53^ Given that $l_{p}$ is not convex and lacks Lipschitz continuity, typically necessitating supplementary smoothing terms in optimization problems to prevent singularity, an alternative non‐convex yet Lipschitz continuous norm, namely the $l_{1-2\;}$ norm, was introduced.^54^ $l_{1-2\;}$ matrix norm is defined as follows:

(2) $$∥U{∥}_{2,1-2}=∥U{∥}_{2,1}-∥U{∥}_{2,2}=∥U{∥}_{2,1}-∥U{∥}_{F}$$

where $∥.{∥}_{F}$ is Frobenius norm.

### 3.5 Propose method: EPCA

Assume input data is $X\in R^{p\times n}$. The optimization function of enhanced PCA (EPCA) is formulated as follows:

(3) $$\min_{U,V}\frac{1}{2}{∥X-UV^{T}∥}_{F}^{2}+\lambda \;\Omega \left(U\right)+\alpha \;\Omega \left(V\right)\;\;s.t\;V^{T}V=I$$

where Ω(U) and Ω(V) are the regularization of principal directions matrix and regularization of principal components matrix, respectively. Parameters λ and α are regularization coefficients.

Regularization is frequently applied on the principal components matrix V in regularized PCA,^55^ Nevertheless, the reconstruction error can be directly affected by principal directions matrix U. Since $X\approx UV^{T}$, we can represent each element of X by linear combination of $V^{T}$ with weight U. Therefore, the matrix U can play an important role in the representation of data in subspace.

The formulation of the objective function for proposed method is expressed as follows:

(4) $$\begin{array}{c} \min_{U,V}\frac{1}{2}∥X-UV^{T}{∥}_{F}^{2}+\lambda \;∥U{∥}_{2,1-2}\\ +\alpha \;\mathrm{Tr}\left(V^{T}HV\right)\;\;s.t\;V^{T}V=I \end{array}$$

where Tr is the trace of matrix and H is Hessian graph matrix.

To solve the (4), we solve for variables U and $V^{T}$ separately when another variable is fixed.

#### 3.5.1 Solving for U by fixing $V^{T}$

We have following objective function:

(5) $$\min_{U}\frac{1}{2}∥X-UV^{T}{∥}_{F}^{2}+\lambda \;∥U{∥}_{2,1-2}$$

As we know $∥U{∥}_{2,1-2}=∥U{∥}_{2,1}-∥U{∥}_{2,2}$ and it is not convex. This nonconvexity difficulty is solved using concave–convex procedure (CCCP) technique.^56^

##### Concave‐convex procedure (CCCP)

If we have two convex functions f(x) and g(x), h(x)≔f(x)−g(x) is convex if and only if the g(x) is affine, otherwise it is nonconvex. To address this concern, CCCP is applied to linearize the concave term (g(x)) in h(x) at iteration k th.^39^, ^57^CCCP solves problem h(x) iteratively as follows:

(6) $$∥U{∥}_{2,1-2}=∥U{∥}_{2,1}-\langle U,\nabla ∥U^{k}{∥}_{F}\rangle$$

where ∇ is gradient and $\nabla ∥U^{k}{∥}_{F}$ shows the value at iteration k ‐th and it is calculated as follows:

(7) $$\nabla ∥U^{k}{∥}_{F}=\left\{\begin{array}{c} \frac{U^{k}}{∥U^{k}{∥}_{F}},\;\;\;\;\mathrm{if}\;U^{k}\neq 0\;\\ 0,\;\;\;\;\;\;\;\;\;\;\;\mathrm{Otherwise} \end{array}\right.$$

The mathematical details of CCCP can be found in the S‐CCCP supplementary data.

Therefore, (5) can be written using a CCCP trick as follows:

(8) $$J=\min_{U}\frac{1}{2}{∥X-UV^{T}∥}_{F}^{2}+\lambda \left(Tr\left(U^{T}QU\right)-\langle U,g\rangle \right)\;$$

where J is an objective function, $Q=diag(\frac{1}{4∥u^{i}{+\varepsilon ∥}_{2}^{2}})$, $g=∥U{{∥}_{F}}^{-1}U$ and ε is a small value to guarantee a non‐zero value in denominator of Q to stabilize the fraction ($u^{i}$ represents i ‐row of matrix U.).

Therefore, we obtained the optimal solution of U as follows:

(9) $$\frac{\partial J}{\partial U}=-\left(X-UV^{T}\right)V+2\lambda QU-g=0$$

Thus $U={\left(I+2\lambda Q-∥U{{∥}_{F}}^{-1}I\right)}^{-1}XV$.

#### 3.5.2 Solving for V

Let us define A = $I+2\lambda Q-∥U{{∥}_{F}}^{-1}I$ and substitute $U=A^{-1}XV$ into (4). Where, I is identity matrix.

Then, the optimal V is computed by solving following problem:

(10) $$\min_{V}\frac{1}{2}{∥X-A^{-1}XVV^{T}∥}_{F}^{2}+\alpha \;\mathrm{Tr}\left(V^{T}HV\right)\;\;s.t\;V^{T}V=I$$

Equation (10) is converted to following problem using trace properties (circularity property):

(11) $$\min_{V}\;Tr\left(V^{T}\left(X^{T}\left(2A^{-1}+A^{-2}\right)X+2\alpha H\right)V\right)\;\;s.t\;V^{T}V=I$$

By considering $\;\Psi =X^{T}\left(2A^{-1}+A^{-2}\right)X+2\alpha H$, we have

(12) $$\min_{V}\;Tr\left(V^{T}\Psi V\right)\;\;s.t\;V^{T}V=I$$

Problem (12) is an eigen problem and solution is first k eigenvectors of matrix ψ corresponding to k smallest eigenvalues of matrix Ψ.

The proposed method is summarized in algorithm 1.

ALGORITHM 1 **Iterative update process of EPCA.**

**Table:** 

**Input**: $X_{1},$ $X_{2}$, α, and λ: $X_{1}$: CT radiomic features $X_{2}:$ QUS radiomics features $X=[X_{1}\;X_{2}]$: concatenating CT radiomic features and QUS radiomics features. **Stage 1**: Construct Hessian matrix H. **Stage 2**: Initialize the U and V. **Repeat** $U^{t+1}={\left(I+\lambda Q-\frac{1}{2}∥Q^{t}{{∥}_{F}}^{-1}I\right)}^{-1}XV^{t}$ $Q^{t+1}=diag\left(\frac{1}{4∥u^{t}{+\varepsilon ∥}_{2}^{2}}\right)$ $V^{t+1}=eigenvectors\;\left(\Psi \right)$ responding to small eigenvector of $\psi^{t}$ **Until convergence** **output**: Fused features

Principal components are eigenvectors of matrix $\Psi =\{\psi_{1},\psi_{2},\text{…},\psi_{k}\}$. Principal components of Ψ were sorted in descending form ($\psi_{1}>\psi_{2}>\cdots >\psi_{k}$) based on eigenvalues of Ψ. The top principal components were selected, and the normalized data was projected onto these components.

### 3.6 Response prediction using machine learning classifiers

Treatment response prediction in the work here was a binary classification task. Two classifiers were applied including KNN methodology and a support vector machine (SVM) to discriminate complete responder and partial responder patient groups. A radial basis function (RBF) kernel was used for the SVM. The hyperparameters of SVM (parameter “C”, which is a trade‐off between the complexity of a model and the number of non‐separable samples, and gamma which is the width of radial basis function (RBF) kernel) and KNN (number of neighbors) were optimized using a grid‐search. The performance of the classifiers was evaluated by their accuracy (ACC), F1‐score (F1), balanced accuracy (BACC), Sensitivity (*S*_n_), and Specificity (*S*_p_).

Leave‐one‐patient‐out (LOPO) methodology was applied to spilt data into a training set and test‐set. Training data was normalized using the z‐score (mean subtraction and standard deviation normalization) to have data with zero mean and one standard deviation. The synthetic minority oversampling technique (SMOTE) was utilized to address the challenge of imbalanced data.^58^ SMOTE was applied only on the training set. Fivefold cross‐validation was applied on training set to tune hyper parameter of SVM and KNN. Additionally, we applied bootstrap on LOPO predictions to evaluate variability in LOPO. By applying LOPO, the model has contribution of each patient in both training and evaluation.

All experiments were implemented using the Matlab 2020b programming environment in a machine having a 1.5 GHz Intel(R) Core (TM) i7‐1065G7 CPU and 16 GB RAM.

In order to evaluate the effectiveness of the proposed method EPCA, five comparative techniques were used.

These techniques are described below:

1. Feature selection (mRMR‐SFS): In this technique, features in training‐set were ranked by mRMR and then sequentially feature selection (SFS) was applied to obtain the best performance.
2. PCA: Traditional PCA was applied to fuse the features.
3. Kernel PCA (KPCA): KPCA was applied to fuse the features and extract the non‐linear relationships in data.
4. Autoencoder:^59^ This technique applied an autoencoder to fuse the features and considered the bottleneck of autoencoder as fused features.
5. Canonical correlation analysis fusion (CCAF^60^): Canonical correlation analysis (CCA) was used to measure the association between two variables. CCA aims to find a linear combination with aim of maximizing the pair‐wise correlations between the two variables. QUS radiomics features and CT radiomics features were two variables which were fused by CCAF. Fused features are a summation of canonical variates of CCA.

These five comparative techniques include feature selection, traditional PCA, an enhanced PCA technique, deep learning using autoencoders, and CCA for fusing two distinct datasets. Feature selection is a preprocessing step aimed at selecting the most relevant features for training machine learning models. The mRMR‐SFS technique ranks features and identifies the optimal number of features for model training. However, mRMR‐SFS concatenates features from different imaging modalities in a straightforward manner. Traditional PCA served as a baseline to demonstrate the performance improvement achieved by Enhanced PCA (EPCA). Autoencoder methodology, as a deep learning approach, provided a robust comparison between EPCA and deep learning methods. The architecture of autoencoder can be found in Ref 59.

### 3.7 Statistical testing

The two‐side *t*‐test was applied on principal components to assess whether they were statistically significant.

**FIGURE 5:**
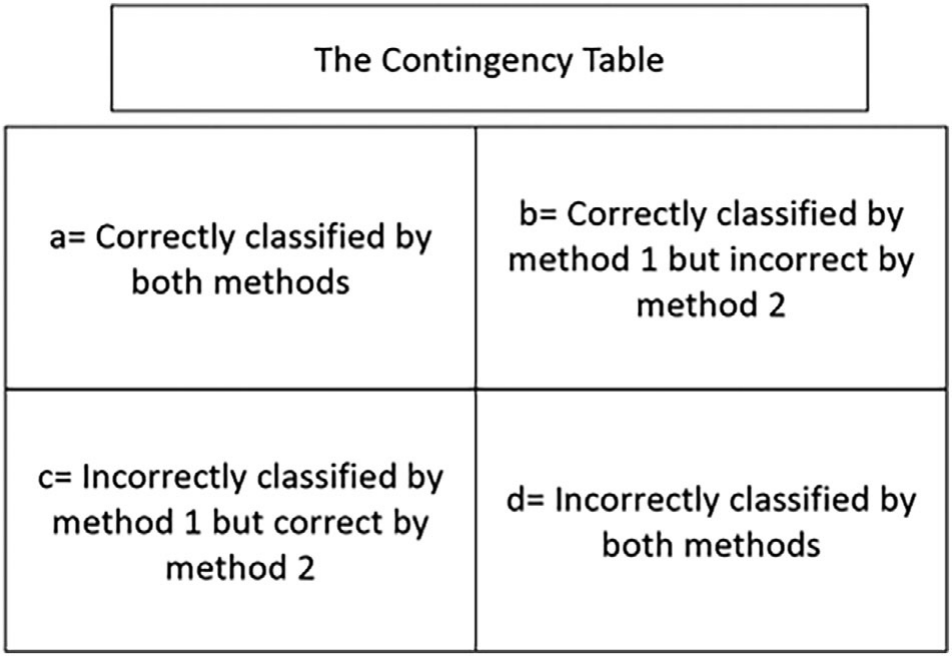
The contingency table for the McNemar test.

In relation to the comparison of classification performance, the McNemar test is an effective technique.^61^ The McNemar test was based on a cross‐tabulation of the classifier accuracy as shown in Figure 5. We applied the McNemar test to compare the results of EPCA with mRMR‐SFS, PCA, KPCA, autoencoder, and CCAF.

The McNemar test statistic chi‐squared ($\chi^{2}=\frac{{\left(b-c\right)}^{2}}{b+c}$) were computed between EPCA and each of comparative techniques. Two classification results would be declared different at level 0.05 if $\chi^{2}>3.84$.^62^

## 4 RESULTS

Among 71 enrolled patients, *n = *66 (93%) were male. The mean age of the patients was 58.9 years (standard deviation = 10.2) at the time of diagnosis. Patients *n = *61 (86%) were treated with chemotherapy along radiation therapy and *n = *10 (14%) patients were treated only by radiation therapy. There were *n* = 25/71 (35%) CR and *n* = 46/71 (65%) PR amongst 71 patients. 546 radiomics features were determined from CT (70 features) and QUS (476 features) data. Patient details, diseases, and treatment characteristics are summarized in Table 1 and per patient clinical details are presented in Table S1. No statistically significant differences were found in comparison between CR and PR patients.

**TABLE 1:** Summary of patients’ cohort involved in this study.

Patient characteristics	*n* (%)
**Patient and clinical characteristics *n* = 71 (all subjects)**	
Age (years) ‐ Mean (Std)	58.9 (10.2)
Gender ‐ Male ‐ Female	66 (93) 5 (7)
Smoking status: ‐ Smoker ‐ Non‐smoker ‐ Unknown	47 (66.2) 23 32.4) 1 (1.4)
Drinking status: ‐ Drinker ‐ Non‐drinker ‐ Unknown	50 (70.4) 15 (21.1) 6 (8.4)
**Tumor status**	
Primary tumor(T): ‐ T1 ‐ T2 ‐ T3 ‐ T4 ‐ Unknown	4 (5.6) 23 (32.3) 7 (9.8) 14 19.7) 23 (32.4)
Histological type: ‐ Squamous cell carcinoma ‐ Small cell carcinoma ‐ Nasopharyngeal carcinoma	66 (92.9) 1 (1.4) 4 (5.6)
HPV status: ‐ p16 positive ‐ p16 negative ‐ Unknown	40 (56.3) 2 (2.8) 29 (40.8)
**Treatment characteristics**	
Chemotherapy ‐ Cisplatin ‐ Carboplatin ‐ Carboplatin + etoposide ‐ Cetuximab No chemotherapy	63 (88.7) 56 (78.8) 5 (7) 1 (1.4) 1 (1.4) 8 (11.2)
**Post treatment (3‐month assessment from MRI)**	
Complete responder—locoregional control (CR) Partial responder—locoregional failure (PR)	25 (35.2) 46 (64.8)

Abbreviation: Std, Standard Deviation.

The proposed EPCA method demonstrated the best performance in discriminating PR and CR outcomes using a SVM classifier. Specifically, EPCA obtained the best performance using a SVM classifier with an 81% balanced accuracy (Sensitivity (*S*_n_) = 79±2%, Specificity (*S*_p_) = 84 ±2%. Accuracy (ACC) = 82 ±2%, balanced accuracy (BACC) = 81 ±2, F1‐score (F1) = 80±2%, AUC = 82 ±2%). For the SVM classifier, mRMR‐SFS had poorer performance (Sensitivity (*S*_n_) = 79±2%, Specificity (*S*_p_) = 76±2%. Accuracy (ACC) = 78±2%, balanced accuracy (BACC) = 77±2, F1‐score (F1) = 76±2%, AUC = 78±2%) and autoencoder results were worse (Sensitivity (*S*_n_) = 62±3%, Specificity (*S*_p_) = 80 ±  3%, Accuracy (ACC) = 72±  3%, balanced accuracy (BACC) = 71±3, F1‐score (F1) = 71 ±  3%, AUC = 72±  3%). Tables 2 and 3 show the results of the proposed method and comparative methods using a KNN classifier and a SVM classifier, respectively.

**TABLE 2:** The performance of classification using KNN.

	%S_n_ mean ± std	%S_P_ mean ± std	%ACC mean ± std	%BACC mean ± std	%F1 mean ± std	%AUC mean ± std
mRMR‐SFS	71 ± 3	71 ± 3	71 ± 3	71 ± 3	70 ± 3	71 ± 3
PCA	54 ± 2	61 ± 2	59 ± 2	57 ± 2	55 ± 2	59 ± 2
KPCA	54 ± 2	60 ± 2	60 ± 2	58 ± 2	55 ± 2	59 ± 2
Autoencoder	58 ± 3	78 ± 3	69 ± 3	68 ± 3	67 ± 3	68 ± 3
CCAF	57 ± 3	77 ± 3	69 ± 3	67 ± 3	65 ± 3	67 ± 3
Proposed method: EPCA	72 ± 2	75 ± 2	74 ± 2	73 ± 2	72 ± 2	74 ± 2

**TABLE 3:** The performance of classification using SVM.

	%*S*_n_ mean ± std	%*S*_P_ mean ± std	%ACC mean ± std	%BACC mean ± std	%F1 mean ± std	%AUC mean ± std
mRMR‐SFS	79±2	76 ± 2	78 ± 2	77 ± 2	76 ± 2	78 ± 2
PCA	59 ± 4	71 ± 4	68 ± 4	64 ± 4	63 ± 4	67 ± 4
KPCA	60 ± 3	71 ± 3	68 ± 3	65 ± 3	63 ± 3	68 ± 3
Autoencoder	62 ± 3	80 ± 3	72 ± 3	71 ± 3	71 ± 3	72 ± 3
CCAF	61± 2	81 ± 2	73 ± 2	71 ± 2	69 ± 2	72 ± 2
Proposed method: EPCA	79 ± 2	84 ± 2	82 ± 2	81 ± 2	80 ± 2	82 ± 2

We combined EPCS with SVM and KNN, the SVM classifier had better performance overall than KNN. The RBF kernel was utilized for SVM, enabling the classification of samples that are not linearly separable. Additionally, SVM is powerful algorithm which can be used to extract pattern of complex data, and it is robust to overfitting challenge since it is worked based on risk minimization. We added components sequentially to classifier to obtain the optimum number of components. The best performance was obtained using six eigenvectors of Ψ and SVM classifier. A two‐side *t*‐test was applied in order to assess the statistical significance of the six principal components.

A tow‐side statistical test was applied on top six principal components to assess these components. Table 5 presents the results of the *t*‐test evaluation between patients with CR and patients with PR for the six principal components (eigenvectors of Ψ). We applied the *t*‐test with Bonferroni correction to perform the multi‐comparison correction.^63^ Based on Table 4, $\psi_{4}$ was statistically significant after applying multi‐comparison correction.

**TABLE 4:** The results of two‐side *t*‐test on top six principal components.

Principal components	*p*‐value
$\psi_{1}$	0.10
$\psi_{2}$	0.04
$\psi_{3}$	0.19
$\psi_{4}$	0.001^†^
$\psi_{5}$	0.09
$\psi_{6}$	0.12

^†^ : Shows statistically significant.

McNemar's test was subsequently applied in order to compare the proposed method with others in terms of effective size. Table 5 presents the results of McNemar's test of comparison between the EPCA methodology and comparative techniques (mRMR‐SFS, PCA, KPCA, autoencoder, and CCAF).

**TABLE 5:** The results of McNemar's test comparing EPCA and comparative techniques. The performance of SVM classifier was use to fill out the associated contingency table.

Method	Chi‐square value of McNemar's test
mRMR‐SFS	1.09
PCA	6.5^†^
KPCA	6.2^†^
Autoencoder	4.8^†^
CCAF	4.1^†^

^†^ : Statistically significant.

Based on Table 5, the results of McNemar's test, the output of EPCA with SVM classifier was statistically significant for all comparative techniques, with the exception of mRMR‐SFS.

The top features based on loading vector (contribution of each original variable to principal components) were QUS‐GLRLM‐gray‐level‐nonuniformity‐normalized, QUS‐GLSZM‐zone‐entropy, CT‐GLCM‐correlation, QUS‐GLDM‐dependence‐variance, QUS‐GLRLM‐low‐gray‐level‐run‐emphasis, and QUS‐GLCM‐sum‐square.

### 4.1 Parameter sensitivity

The proposed EPCA has two parameters, λ and α. Parameter λ controls the sparsity and is a trade‐off coefficient between sparsity regularization and reconstruction error. Parameter α is a regularization coefficient for structure learning to control the contribution of the Hessian regularization term. Parameters λ and α were tuned using a grid search strategy in the range of $\left\{10^{-6},10^{-4},10^{-2},\text{…},10^{6}\right\}$.

### 4.2 Sparse regularization analysis

In this study, the $l_{2,1-2}$ ‐norm was applied to obtain a sparse solution. As an ablation study, in this section, the $l_{2,1-2}$ ‐norm was compared with a $l_{2,1}$ ‐norm, and Table 6 shows the classification results. For the $l_{2,1}$ ‐norm, the PCA is formulated as follows:

(13) $$\begin{array}{c} \min_{U,V}\frac{1}{2}∥X-UV^{T}{∥}_{F}^{2}+\lambda \;∥U{∥}_{2,1}\\ +\alpha \;\mathrm{Tr}\left(V^{T}HV\right)\;\;s.t\;V^{T}V=I \end{array}$$

**TABLE 6:** The classification results using SVM classifier for EPCA with $l_{2,1-2}$ ‐norm and EPCA with $l_{2,1}$ ‐norm (EPCA has Hessian regularization).

	%*S*_n_ mean ± std	%*S*_P_ mean ± std	%ACC mean ± std	%BACC mean ± std
$l_{2,1-2}$ ‐norm	79± 2	84± 2	82 ± 2	81 ± 2
$l_{2,1}$ ‐norm	75 ± 2	81 ± 2	79± 2	78 ± 2

Based on Table 6, the $l_{2,1-2}$ ‐norm had better performance than the $l_{2,1}$ ‐norm. Specifically, an improvement can be seen in sensitivity. Specifically, EPCA with $l_{2,1-2}$ ‐norm obtained *S*_n _= 79 ±  2% (ACC = 82±2%, BACC = 81 ±2%, *S*_P _= 84 ±  2%) and EPCA with $l_{2,1}$ ‐norm obtained *S*_n _= 75±2% (ACC = 79 ±2%, BACC = 78 ±  2%, *S*_P _= 81±  2%). Based on results, the more sparsity of $l_{2,1-2}$ ‐norm led to improvement of classification results.

### 4.3 Hessian graph analysis

The Laplacian graph is commonly used to preserve the local topology of samples and the geometrical relationships of data in the low embedding space However, the Hessian graph was employed here. Table 7 shows the comparison between EPCA with the Hessian graph and EPCA with the Laplacian graph. The $l_{2,1-2}$ ‐norm was used as sparsity regularization to compare EPCA with the Hessian graph and EPCA with the Laplacian graph.

**TABLE 7:** The classification results using SVM classifier for EPCA with Hessian graph and EPCA with Laplacian graph.

	%*S*_n_ mean ± std	%*S*_P_ mean ± std	%ACC mean ± std	%BACC mean ± std
Hessian graph	79± 2	84± 2	82 ± 2	81± 2
Laplacian graph	73±3	85 ±3	80±3	77 ±3

Based on Table 7, the performance of the SVM classifier using the Hessian graph was better than that of the Laplacian graph for predicting CR and PR. Specifically, the Hessian graph had considerably better sensitivity than the Laplacian graph. The Hessian graph achieved an accuracy of 82±  2% (*S*_n_ = 79±2%, BACC = 81 ±  2%, *S*_P_ = 84 ±2%), while the Laplacian graph achieved an accuracy of 80 ±3% (*S*_n_ = 73 ±3%, BACC = 77 ±3%, *S*_P_ = 85 ±3%).

### 4.4 Comparing with single imaging modality

In this section, we compared feature‐level fusion of CT and QUS with CT and CUS as single modality imaging. Table 8 shows the prediction results of feature‐level fusion of CT and QUS, and CT and CUS as single modality imaging, using mRMR‐SFS.

**TABLE 8:** The classification results using the SVM classifier for feature‐level fusion of CT and QUS (EPCA), as well as for CT and QUS single modalities, are presented.

	%*S*_n_ mean ± std	%*S*_P_ mean ± std	%ACC mean ± std	%BACC Mean ± std
QUS	76± 2	81± 2	78± 2	77± 2
CT	70 ±3	68±3	69 ±3	69±3
EPCA^*^	79± 2	84 ± 2	82± 2	81 ± 2

* : Feature level fusion of CT and QUS.

Based on the results from Table 8, feature‐level fusion using EPCA achieved the best performance. The BACC of EPCA is 12% and 4% higher than that of CT and QUS, respectively. These results indicate that feature‐level fusion leads to a considerable improvement in predicting PR and CR for patients with H&N cancer.

### 4.5 Ablation study

In this subsection, methods of EPCA and robust PCA (rPCA) were compared.^64^ rPCA was proposed to overcome any outlier effects. rPCA was modeled as follows:

(14) $$\min∥Z{∥}_{*}+\lambda ∥S{∥}_{1}\;\;\;s.t\;\;X=Z+S$$

where Z and S are low‐rank components and sparse components, respectively. $∥.{∥}_{*}$ is the nuclear norm (sum of singular values) and λ is a trade‐off parameter between low‐rankness and sparsity. The alternating direction method of multipliers (ADMM) was applied to solve the problem (14) which incorporates both the primal and dual variables in optimization.

To apply rPCA for outcome prediction of H&N; we first applied rPCA and then applied conventional PCA on the matrix Z to extract fused features. Table 9 shows the performance of EPCA in comparison with rPCA when the SVM classifier was used to predict response.

**TABLE 9:** Classification results using the SVM classifier for EPCA and rPCA.

	%*S*_n_ mean ± std	%*S*_P_ mean ± std	%ACC mean ± std	%BACC mean ± std
EPCA	79± 2	84 ± 2	82 ± 2	81 ± 2
rPCA	75± 2	78 ± 2	77 ± 2	76 ± 2

Based on Table 9, EPCA outperformed rPCA with a 5% difference in accuracy and balanced accuracy each. rPCA was designed to ameliorate the effect of noise and outliers. It cannot preserve the manifold information of data in subspace, which is playing vital role to preserve the geometrical information of data.

Additionally, we compared EPCA with $l_{2,1}$ ‐norm constrained graph Laplacian PCA.^65^ $l_{2,1}$ ‐norm constrained graph Laplacian PCA is formulated as follows:

(15) $$\min_{U,V}\frac{1}{2}∥X-UV^{T}{∥}_{F}^{2}+\lambda \;∥U{∥}_{2,1}+\alpha \;\mathrm{Tr}\left(V^{T}LV\right)\;\;s.t\;V^{T}V=I$$

where L is Laplacian matrix. The detail of the Laplacian graph and how it is constructed can be found in the S‐Laplacian Graph Matrix supplementary data. Table 10 shows the performance of EPCA in compared with $l_{2,1}$ ‐norm constrained graph Laplacian PCA when the SVM classifier was used to predict response. The solution of (15) can be found in the S‐$l_{2,1}$ ‐norm constrained graph (supplementary data S‐$l_{2,1}$).

**TABLE 10:** The classification results using SVM classifier for EPCA and $l_{2,1}$ ‐norm constrained graph Laplacian PCA.

	%*S*_n_ mean ± std	%*S*_P_ mean ± std	%ACC mean ± std	%BACC mean ± std
EPCA	79± 2	84 ± 2	82 ± 2	81 ± 2
$l_{2,1}-L-$ PCA^*^	76 ±3	81 ±3	79 ±3	78 ±3

$l_{2,1}-L-$ PCA^*^:$l_{2,1}$ ‐norm constrained graph Laplacian PCA.

Based on Table 10, EPCA outperformed $l_{2,1}$ ‐norm constrained graph Laplacian PCA. This result indicated the effectiveness of Hessian graph matrix and $l_{2,1-2}$.

## 5 DISCUSSION

In this study, EPCA was proposed in order to fuse quantitative CT and QUS data at the feature level. The work used 546 features from 71 patients, which indicates that an underdetermined system of equations was encountered, leading to an ill‐posed condition. In such situations, dimensionality reduction becomes a necessary preprocessing step for building a predictive model. Whereas feature selection offers better interpretability than feature extraction, given the presence of two types of imaging modalities, feature selection here ideally must follow a multi‐view clustering approach. Since the features originate from two different sources (QUS and CT radiomics features), the naive approach of directly concatenating all features and considering them as single‐modality features is potentially inadequate. That approach may overlook the divergence among imaging modalities, resulting in a deterioration of information.^66^ Additionally, using multi‐view clustering techniques is challenging, as there is a view weight coefficient applied to control the distribution of each view (imaging modality).^67^ Obtaining the optimal solution for the view weight coefficient is a challenge for multi‐view clustering, and the performance of feature selection can be directly affected by this coefficient. Therefore, feature fusion is an effective approach to consider that neither concatenates all features nor faces the challenge of the view weight coefficient. To build machine learning model, we employed LOPO to have maximum number of samples in training phase and bootstrap sampling to estimate the mean and standard deviation of performance metrics. However, LOPO can lead to optimistic estimates due to high variance across folds, and the bootstrap may fail to completely capture the variability in real independent external populations. In our study, bootstrap resampling was applied only to the fixed LOPO predictions. It means the model was not retrained during the resampling process. Consequently, the reported variance shows the patient‐level prediction uncertainty and does not account for variation introduced by model retraining or hyperparameter tuning. Nevertheless, future studies could address this limitation by employing nested cross‐validation or repeated LOPO training to better capture these additional sources of uncertainty. In the work here, adding two regularizations (the $l_{2,1-2}$ ‐norm and Hessian matrix) improved the performance of PCA algorithm. $l_{2,1}$ ‐norm is the only slack variable, and it is not sufficiently sparse^68^ and $l_{2,p}$ (0<p<1) is neither convex and nor Lipschitz continuous.^57^ Whereas $l_{2,1-2}$ ‐norm is sparser than $l_{2,1}$ ‐norm and it is Lipschitz continuous in contrast to $l_{2,p}$. Furthermore, the Hessian graph matrix has better performance than the Laplacian graph matrix to preserve geometrical information of samples in embedded space.^69^ Additionally, Kim et al.^38^ using digit and figure datasets showed that Hessian graph regularization can preserve the geometry of data considerably stronger than Laplacian graph regularization. In terms of dimensionality reduction, other work showed that Hessian regularization has better performance than Laplacian graph regularization to preserve the geometrical information of data.^70^ In Sections 4.2 and 4.3, we compared EPCA with $l_{2,1}$ sparse and Laplacian graph, the results in Tables 6 and 7 show the effectiveness of $l_{2,1-2}$ ‐norm and Hessian matrix graph. Additionally, we compared EPCA with $l_{2,1}$ ‐norm constrained graph Laplacian PCA.^65^ Based on Table 10, EPCA had better performance (accuracy = 82±2%) than $l_{2,1}$ ‐norm constrained graph Laplacian PCA (accuracy = 79±3%). EPCA utilizes the Hessian graph matrix, which can preserve the topological structure of the data more effectively than the $l_{2,1}$ ‐​norm‐constrained graph Laplacian PCA. The Laplacian graph matrix obtains a solution based on a constant geodesic function; as a result, it fails to effectively preserve the geometric information of the data.^38^ In contrast, the Hessian matrix captures linear changes based on geodesic distance, leading to more robust preservation of the topological structure. Therefore, Hessian graph preserves local geometry of the data manifold more efficient than Laplacian graph.

Additionally, the $l_{2,1}$ ‐norm is not sufficiently sparse, making the solution less interpretable and sparse. Although the $l_{2,1-2}$ ‐norm is non‐convex, it is Lipschitz continuous and provides sparser solutions than the $l_{2,1}$ norm. The CCCP technique was employed to address the challenge of non‐convexity. The superiority of the $l_{1-2}$ norm over the $l_{1}$ ‐norm in the context of sparse representation learning has been demonstrated.^56^

In terms of discriminating between PR and CR, PCA obtained the lowest rank among the comparative methods in performance. PCA cannot preserve the geometric information of data and is very sensitive to noise and outliers, which is why its performance may not be as good as that of other techniques. Since PCA does not have any regularization function and it captures direction of maximum variance, it can consider the noisy component as the top one. That is why EPCA (accuracy = 82±  2%) obtained 14% higher accuracy than PCA (accuracy = 68±4%). KPCA inherits the drawbacks of PCA, and loss of orthogonality is possible. Principal components must be orthogonal to each other, and we preserved this in EPCA by having $V^{T}V=I$ constraint and Table 3 showed the effectiveness of EPCA in comparison with KPCA. Additionality, KPCA lacks interpretability. The CCAF method suffers the singularity challenge of matrix covariance of each variable, and the performance of fusion can be affected. CCAF is highly sensitive to the dimensionality of the data and faces significant challenges when applied to small datasets. Additionally, CCAF typically shows stable performance when the sample size is much larger than the number of features. In terms of performance, CCAF obtained 73±2% accuracy which is 9% less than EPCA accuracy. EPCA had better performance than CCAF to predict minor samples and Table 3 supports this such that EPCA had 81±2% balanced accuracy and CCAF had 71±2% balanced accuracy. Loss of information in the encoding process and choosing architecture and hyperparameters are the main challenges for an autoencoder. The architecture of autoencoder networks plays a significant role in extracting an informative representation of features. mRMR‐SFS obtained the second‐best performance. It ranks the features and adds a new feature to the previous in each iteration. If there are four features $F_{1}$, $F_{2}$, $F_{3},$ and $F_{4}$ with importance $F_{1}>$ $F_{2}>F_{3}>F_{4}$. The top‐2 features are $F_{1}$ and $F_{2}$ and mRMR‐SFS considers these two features. However, Nie et al.^68^ showed that the combination of $F_{1}$ and $F_{4}$ could provide a better separation in embedded space. This is the main challenge of mRMR‐SFS as a classic feature selection technique. The combination of features in subspace is more important than rank of features. Since mRMR is a supervised, filter‐based feature selection technique, it does not take classifier performance into account during feature selection. Additionally, mRMR does not incorporate adaptive weighting between relevance and redundancy, instead assigning a fixed, equal weight to both.

In comparing with single imaging modality, results in Table 8 showed that the fusion of CT and QUS is an effective approach to improve the performance of predictive model. Based on Table 8, fusion of QUS and CT using EPCA obtained 82±2% accuracy in comparison of 78±2% accuracy using only QUS and 69±3% accuracy using only CT.

A statistical test on principal components of EPCA showed that the first principal component is statistically significant. Additionally, McNemar's test results in Table 5 showed that the output of proposed method in terms of effect sizes is statistically significant in comparison with PCA, KPCA, autoencoder, and CCAF, and only it is not statistically significant in comparison with mRMR‐SFS.

EPCA is a feature extraction‐based technique that suffers from limited interpretability, which is important for clinical studies. Additionally, although standardization protocols were applied to the images, variability in image acquisition may still affect the results.

In the context of H&N cancer outcome prediction, Diamant et al.^71^ applied CNN directly on CT images of 300 patients with head and neck squamous cell carcinoma to predict outcome. They used an Inception‐V3 pretrained network to predict outcome. They could use only three slices of each image since they applied a pretrained network and in such a situation the topology information of tumor cannot be preserved. A second issue in that work was that the work did not unfreeze the last layer on Inception‐V3 and then all convolutional filter weights came from ImageNet dataset which was not medical dataset. In other work, Huynh et al^72^ did a comparison between machine learning with radiomics features and CNN to predict outcomes of H&N cancer treatment using FDG PET/CT images and clinical data. Teo et al.^73^ applied machine learning and ensemble feature selection on clinicopathologic and dosimetric data to identify risk of head and neck cancer treatment‐related lymphedema. They applied an ensemble feature selection technique to identify features associated with lymphedema incidence. The proposed method here rather fused radiomics features and then applied machine learning classifier.

### 5.1 Limitations

There are four main limitations in our study. The dataset here is small and therefore the generalizability of classifier is not fully certain, and the probability of overfitting could be increased. The second constraint pertains to the imbalance between male and female ratios. Whereas there is a considerable gender imbalance, it is important to highlight that the incidence of H&N cancer diagnoses is notably higher in males. This is evident in a 25‐year examination of cancer prevalence in Canada, where, out of approximately 48 000 total H&N cancer cases, around 70% (∼35 000) were identified in males.^74^ The work did not take into account HPV status given the small number of patients and was an attempt to identify radiomic features exclusive of classic clinical features. The third limitation is the lack of dataset diversity. All data were obtained from a single institution. Although the same imaging protocol was used for all patients, using multi‐institutional datasets would enhance the generalizability of the model by capturing a wider range of patient demographics, imaging devices, acquisition protocols, and clinical practices. For future studies, publicly available large datasets or multi‐institutional datasets can be used to tackle this challenge. The fourth limitation is the imbalance between patients with and without chemotherapy. There were only eight patients who did not receive chemotherapy, which did not allow us to build a separate machine learning model and compare the two groups including patients with chemotherapy and without chemotherapy, to specifically assess the contribution of chemotherapy in predicting the response to radiation therapy.

## 6 CONCLUSION

Information fusion of CT and QUS imaging modalities in feature level improved the performance of machine learning algorithm to predict treatment response of H&N cancer. EPCA was proposed as an effective algorithm to fuse QUS and CT radiomic features, aiming not only to reduce the dimensionality of the data and lower the risk of overfitting but also to predict complete response and partial response to radiation therapy before treatment initiation in patients with H&N cancer.

## CONFLICT OF INTEREST STATEMENT

The authors declare no conflicts of interest.

## Data Availability

Contact to corresponding author.
